# Addressing power asymmetries in global health: Imperatives in the wake of the COVID-19 pandemic

**DOI:** 10.1371/journal.pmed.1003604

**Published:** 2021-04-22

**Authors:** Seye Abimbola, Sumegha Asthana, Cristian Montenegro, Renzo R. Guinto, Desmond Tanko Jumbam, Lance Louskieter, Kenneth Munge Kabubei, Shehnaz Munshi, Kui Muraya, Fredros Okumu, Senjuti Saha, Deepika Saluja, Madhukar Pai

**Affiliations:** 1 School of Public Health, University of Sydney, Sydney, Australia; 2 Women in Global Health, New Delhi, India; 3 Pontificia Universidad Católica de Chile, Santiago, Chile; 4 PH Lab & Planetary and Global Health Program, St. Luke’s Medical Center College of Medicine—William H. Quasha Memorial, Manila, Philippines; 5 Operation Smile, Accra, Ghana; 6 University of Cape Town, Cape Town, South Africa; 7 The World Bank, Kenya Country Office, Nairobi, Kenya; 8 University of the Witwatersrand, Johannesburg, South Africa; 9 KEMRI-Wellcome Trust Research Programme, Nairobi, Kenya; 10 Ifakara Health Institute, Ifakara, Tanzania; 11 Child Health Research Foundation, Dhaka, Bangladesh; 12 School of Population and Global Health, McGill University, Montreal, Canada

## Abstract

Seye Abimbola and co-authors argue for a transformation in global health research and practice in the post-COVID-19 world.

**Summary points**

- The Coronavirus Disease 2019 (COVID-19) pandemic, the Black Lives Matter and Women in Global Health movements, and ongoing calls to decolonise global health have all created space for uncomfortable but important conversations that reveal serious asymmetries of power and privilege that permeate all aspects of global health.
- In this article, we, a diverse, gender-balanced group of public (global) health researchers and practitioners (most currently living in the so-called global South), outline what we see as imperatives for change in a post-pandemic world.
- At the individual level (including and especially ourselves), we emphasise the need to emancipate and decolonise our own minds (from the colonial conditionings of our education), straddle and use our privilege responsibly (to empower others and avoid elite capture), and build “Southern” networks (to affirm our ownership of global health).
- At the organisational level, we call for global health organisations to practice real diversity and inclusion (in ways that go beyond the cosmetic), to localise their funding decisions (with people on the ground in the driving seat), and to progressively self-decentralise (and so, divest themselves of financial, epistemic, and political power).
- And at both the individual and organisational level, we emphasise the need to hold ourselves, our governments, and global health organisations accountable to these goals, and especially for governance structures and processes that reflect a commitment to real change.
- By putting a spotlight on coloniality and existing inequalities, the COVID-19 pandemic inspires calls for a more equitable world and for a decolonised and decentralised approach to global health research and practice, one that moves beyond tokenistic box ticking about diversity and inclusion into real and accountable commitments to transformative change.

## Introduction

**Fig 1:**
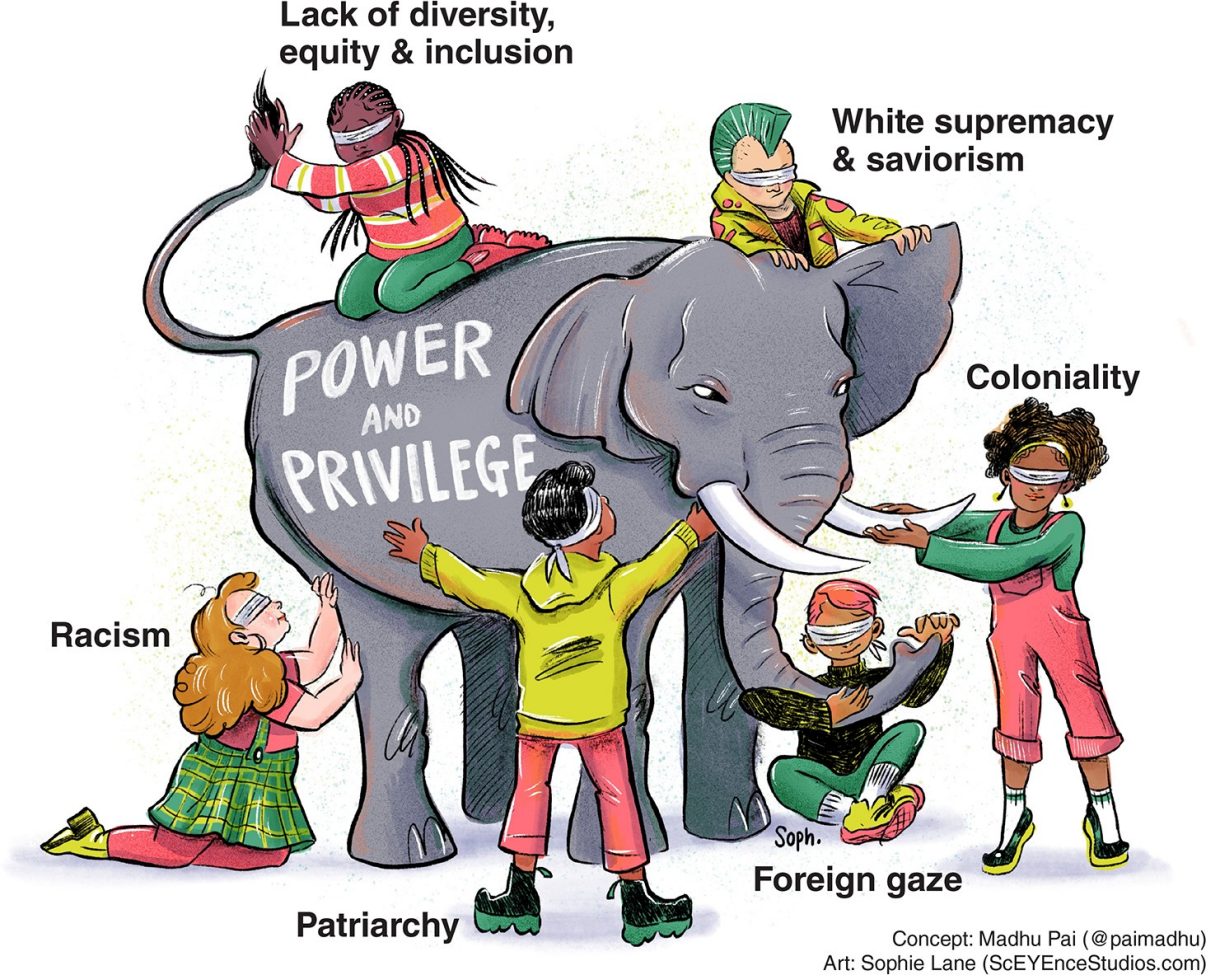
Global health, as currently practiced, has many asymmetries in power and privilege. Coloniality is but one manifestation of supremacy. Therefore, undoing supremacy will require much more than decolonisation. *Image source*: *Madhukar Pai*, *with artwork by Sophie Lane*.

The Coronavirus Disease 2019 (COVID-19) pandemic, the Black Lives Matter movement, and the growing calls to decolonise and address reports of structural racism within humanitarian, development, international aid, and global health agencies are opening doors for uncomfortable but important conversations [1–14]. They are revealing serious asymmetries of power and privilege (Fig 1) that permeate all aspects of global health.

These conversations are happening in many settings, and it is clear by now that they cannot be brushed away. They have increased, as expressed in countless editorials, studies, conferences, and webinars. Even before the COVID-19 pandemic, there was significant but underappreciated discontent among public (global) health practitioners in low- and middle-income countries (LMICs) over discriminatory activities by funding agencies, universities, and individuals from high-income countries (HICs).

However, COVID-19 has put a spotlight on existing inequalities and on processes of coloniality (mind, body, knowledge, and power). It has created conditions for further inequities, with growing populist nationalism and isolationism, widening income disparities, and fractured systems of global cooperation [15,16]. The pandemic continues to enable those with money and power to expand their influence—making decoloniality, solidarity, and distribution of power, knowledge, and resources (e.g., vaccines) even more urgent. The fact that HICs have reserved enough COVID-19 vaccine doses to vaccinate their own population multiple times over is a stark indication of power asymmetry in global health [17].

More than an impasse or a simple opposition within the sector, the real concerns around coloniality and power and privilege hold the potential to reorient the field, in the context of deep uncertainty created by, among other large-scale disruptive processes, the COVID-19 crisis. The impacts of pandemics are unpredictable, and previous country-level epidemic-preparedness indicators have proved inadequate [18], based on faulty assumptions rather than a nuanced understanding of local strengths and weaknesses which can only be understood from the bottom up, and without a supremacist lens on the world [1,7].

In this article, we, a diverse, gender-balanced group of 13 public (global) health researchers, teachers, and practitioners (all born in, and 11 of 13 currently living in the so-called global South), outline our wish list for change in a post-pandemic world—at the individual (including among ourselves), and at the organisational level. Most of us are researchers. Our perspectives, therefore, are more focused on addressing power asymmetries in global health research and education, and ultimately practice.

We recognise that HIC versus LMIC, North versus South, and coloniser versus colonised are crude dichotomies that obscure more than they reveal. Hence, we pay attention in this article to the fact that every grouping has its own internal power hierarchies (as displayed in the Fig 1), with intersectional systemic disadvantages caused, among others, by race, caste, class, ethnicity, gender, and religion. Coloniality is but one manifestation of supremacy. Therefore, undoing supremacy will require much more than decolonisation [19].

## “Decolonising ourselves”: What change do we want to see among ourselves as individuals?

### Emancipate our minds

Many of us need to deliberately decolonise our minds. The most dangerous locus of colonisation is not physical, but our minds [20]. Colonisation was designed to insidiously permeate every aspect of our value judgement as humans. As Ngũgĩ wa Thiong’o observed in his book *Decolonising the Mind*, “the colonial classroom became a tool of psychological conquest in Africa and beyond… and it made the conquest permanent” [21]. Many of us are products of such deliberate and persisting colonial education policies, often reinforced by the higher education many of us have been privileged to receive. It is time to undo the colonial mentality of inferiority that many of us were raised to possess.

To do so, we must build deep and collective awareness of how our colonial histories have shaped our thinking and continue to influence our way of seeing and doing. We must make conscious efforts to unlearn the idea of Western research and knowledge systems, as opposed to local research and traditional/indigenous knowledge systems, as being the only way to advance healthcare or effect change. We must constantly pay attention in our language (for example, use of terms such as “beneficiaries” or “Third world”) and in our daily lives and work, to reject the misguided urge to fix the lives or problems of people who are oppressed or disadvantaged—and instead, use our voice and influence to redistribute power in ways that enable the legitimisation and acknowledgement that marginalised people are the experts of their own lives.

Our role as researchers and practitioners is to be allies and work in solidarity with marginalised people in the process of achieving the changes that they seek. Learning and practicing critical allyship is not only for changing our own behaviours, but also for fundamentally shifting the systems that oppress people [22]. Effective allyship will require us to recognise the privileges, opportunities, resources, and power we have been accorded while others have been overtly or subtly denied them.

Further, we must bring our authenticity, our experiences, our background, our proximity to the work, and the variety of influences that inform our ideas into the spaces in which we engage—turning into strength things that have traditionally been used to make us feel like imposters, e.g., being a woman, or a person of colour, or a local, being locally trained, or not having English as a first language [23]. We must refocus our attention to the local gaze, to local needs, priorities, communities, and decision-makers, so that we are more responsive to those than to external “Requests for Proposals” in our choice of focus. Finally, we must learn from Black, Indigenous, and feminist movements how to shift away from the coloniser’s model of the world, and to help us unlearn, unthink, and undo the logics and doings of coloniality [9,24].

It is an uphill battle to unthink and unlearn the dominant models that for many of us have been easy shortcuts for making sense of the world and making progress in our own education (e.g., some of us earned higher degrees in the global North) and careers (e.g., some of us now work in HICs and in privileged LMIC institutions). These actions require that we are accountable to and support one another as we seek to see the world and our place in it anew.

### Straddle privilege responsibly

Reimagining global health in the post-COVID-19 world requires that we address the intersecting systems of supremacy that continue to limit our ability to achieve equity and justice. Inequities are not only about the needs and concerns of the disadvantaged, but also the systems that create disadvantages. Privilege is complex and relational, [22] as displayed in Fig 1. The social structures that create disadvantages are the same ones that create the advantages from which many of us—including some of the authors of this article—benefit.

To avoid elite capture, we must constantly reflect on our own positionality, behaviour, and unconscious biases, in an ongoing rather than one-off process; lest in the pursuit of equity and justice, we end up perpetuating colonial malpractice. We must be intentional around the complex negotiation that we undertake every day between the different positionalities that we hold. As Senait Fisseha noted, “We are all part of a broken system. Doing good work in the field requires … taking a critical eye to one’s own identity and how one has benefited from a system that oppresses so many others [25].” We need to be able to recognise when we are part of creating the problem and when our choices and actions serve or enable, rather than challenge the status quo that perpetuates othering and dehumanisation.

Dismantling oppressive power requires more than one group of people demanding change. Undoing marginalisation requires more than the marginalised speaking up. Many marginalised groups (to which some of the authors of this paper belong)—e.g., Black, Indigenous, and people of colour (BIPOC), sex workers, migrants and refugees, women and girls, ethnic minorities, people with disabilities, and lesbian, gay, bisexual, transgender, intersex, and questioning (LGBTIQ) people—are systematically denied platforms for political, social, and cultural reasons. But researchers, policy makers, implementers who show solidarity (politically, financially, and emotionally) must allow the marginalised to determine the conditions of engagement in their spaces, recognising, as we straddle spaces, that we must act responsibly and that marginalised people are the experts of their own lives.

Those of us with larger audiences and spaces of influence should disrupt, call out, or shift away from neocolonial practices when we see them in ourselves and in others, including those, who, like us, are working to decolonise global health. Men, in particular, need to “lean out” and create space for women [26]. This requires courage, and it may be costly or uncomfortable to do so. In playing these roles, we must be relentless in practicing reflexivity, submit ourselves to constant challenge, and surround ourselves with people who will demand accountability of us, with a slight nudge or kind reminder when we go astray towards (re-)enacting colonial attitudes and practices.

### Build “Southern” networks

Those of us who have a loud voice in global health should collectively affirm our ownership of the field. We need to claim the space in global health that belongs to us and is proportional to the size of our populations, knowledge, and problems. We need to convert the opportunities we receive into opportunities we give, weave networks of solidarity with peers (e.g., Emerging Voices in Global Health [27] and Women in Global Health with several country chapters [28]), and build collaborations across the global South, without necessarily decreasing “North-South” partnerships. In many global South settings (e.g., in Africa), universities and research institutes are more likely to have global North than in-country or in-continent collaborators, which are essential for solidarity and learning across settings. Even within southern networks, there is a need to engage women, frontline workers, and people with lived experience since they are often invisible in national consultations and committees [29].

Claiming space requires confidence. It requires that we believe that “we can,” and we already hold and have the capacity to produce knowledge. However, our confidence in the potential of our ideas and actions is weakened by the weight of asymmetry that comes with being on the receiving end of (sometimes valuable) support, knowledge, and interventions. Often, we just assume we are not good enough or trained enough and someone with more experience, in a “better” institute in the global North will do something better, without considering the migration of skills from the global South. Sometimes, that belief is foisted on us by colleagues in or funding from the global North. The COVID-19 pandemic has demonstrated the importance of LMIC scientists in generating and using knowledge locally [30,31]. This has shown us that “we can”—we have had no choice but to get on with it (as our “collaborators” were busy with their own response or had to return home quickly), thus boosting confidence that “we can” [31].

But without our governments moving towards self-sufficiency, looking within to maximise the use of local knowledge and capacity, such bursts of confidence will be short lived. Moving from the receiving end of interventions into funding, designing, and implementing local solutions requires local resources and alliances (e.g., through global South networks)—for which we must hold our governments to account [32,33].

To shift the centre of gravity of knowledge production and use, we need domestic funding opportunities and local platforms for knowledge production and use (e.g., academic institutes and journals) that take Indigenous knowledge, local needs, and languages into account, especially because current platforms are often inaccessible (in English, costly, elitist, and distant) [3]. For example, the recent announcements by Springer Nature and Elsevier about high article processing charges is an example of how elitist, exclusive, and exclusionary prestige journals can be [34].

However, we must hold ourselves accountable to avoid elite capture. There is limited value in building new networks and platforms in the global South if they are captured by the local elite like us, such that things remain colonial, and the needs of the centre and the privileged remain prioritised over the periphery and less privileged in policymaking and implementation.

## “Decolonising organisations”: What change do we want to see in global health organisations?

### Real diversity and inclusion

Currently, global health is neither global nor diverse [4,35]. It is therefore not shocking to see growing number of reports of systemic racism, White supremacy, and discrimination in many organisations [12]. Primarily headquartered in HICs (85% in North America, Europe, and Oceania) where major decisions are made, data show that 70% of leaders (CEOs or Board Chairs) in a sample of nearly 200 global health organisations are men, more than 80% are nationals of HICs, and more than 90% were educated in HICs [4].

Global health journals lack diversity [36], and research publications and commissions focused on LMICs are dominated by authors from HICs, who often take the lead and/or senior authorship [37]. *The Lancet* commissions, for example, are dominated by HIC experts, and a vast majority have secretariats based in HIC universities [38]. Awards in global health are mostly given to men and experts from HICs [39].

In short, most global health organisations are run out of HICs, mostly by men, and with staff dominated by people (mostly White) from HICs. And HICs account for a majority of global health spending, and by virtue of controlling the purse strings, they effectively control the global health agenda [40]. If addressing inequities is a central goal of global health, should we continue to entrust that goal to elite HIC institutions who might not reflect the people being served?

All global health organisations (in the global North or global South) must commit to real diversity, equity, and inclusion (DEI) as part of their core mission and ensure that their leadership and staff are diverse and gender balanced without which global health organisations are bound to fail in their mission. Even the most well-intentioned people who claim to not have racist or supremacist biases behave in ways that undermine the expertise and knowledge of (other) local researchers, practitioners, communities, and individuals. Organisations (e.g., universities, bilateral and multilateral agencies, nongovernmental organisations [NGOs], philanthropic organisations, etc.) tend to scapegoat individual staff members when issues of bias arise (e.g., bullying and withholding opportunities). But they need to be held accountable for structures and processes that prevent any form of discrimination by staff—e.g., training for staff, a detailed action plan, and mandates on how the organisation will stand by marginalised communities and how to advocate for their rights.

It is easy to invite people from marginalised groups to join advisory boards or to add African- and Asian-sounding names as coauthors on research articles to please journal editors and peer reviewers. Beyond that, global health organisations need to be held accountable for governance structures and processes that include local partners, and people who are generally underrepresented, in ways that go beyond the cosmetic. For example, reports from communities and local partners may be included in staff performance evaluation. Funding agencies in HICs must make sure they directly fund LMIC organisations that are addressing their own local research priorities. Global health programs in HICs must ensure reciprocity and host trainees and experts from LMICs [41].

These processes of accountability need to be implemented in a context that takes the transformation, liberation, and decolonial agenda seriously [42]. If not, these interventions will become Band-Aids, rather than structural shifts that distribute power and resources. As Themrise Khan warns us, decolonisation is now becoming a “comfortable buzzword for those in the North, driven by the need to not give up power and remain relevant” [43]. The Global South, she emphasises, must end inequality on its own terms—not the North’s.

Global health practice needs a new politics of accountability. Shifting the geography of knowledge from “foreign expertise” to local and Indigenous knowledge holders is part of this new politics [42]. Shifting global health leadership from White-led, White-dominated HIC institutions to BIPOC-led, BIPOC-dominated LMIC institutions is also critical [42]. Drawing on intersectional Black, woman and feminist movements, and Indigenous knowledge systems can facilitate new leadership and organisational practices and theories and processes that centre our humanity through values of radical love, care, compassion, and the redistribution of resources and power.

### Localising funding decisions

Much too often, international donors and funding organisations who come as “saviours,” prefer to fund projects that address their own interests, on their own terms. This, in turn, leads to a waste of resources, loss of local research interest, and lack of trust between grantees and donors.

Even when research or implementation work is focused entirely on LMICs, much of donor funds are given to agencies and institutions in HICs, and HICs hold the purse strings [40]. For example, less than 2% of all humanitarian funding goes directly to local NGOs [44]. About 80% of USAID’s contracts and grants go directly to United States firms [45]. Moreover, 70% of NIH Fogarty grants go to US and HIC institutions [46], and 73% of the total international grant portfolio of the Wellcome Trust supports United Kingdom–based activity [47]. Even with funds are given to LMIC agencies, HIC donors often set the agenda and micromanage the work, leaving little room for LMIC groups to innovate.

Funders need to be held accountable for developing structures and processes for engaging with grantees, for letting grantees guide them on the importance of various projects, and for opening the doors of decision-making to people on the margins, who hold the key to driving change and are closest to the work—i.e., moving away from parachute research and projects towards centring local knowledge and organic processes. LMIC institutions and researchers need to speak out more against parachute research and demand greater control of funding and research output (e.g., publications). They also need to ensure that reciprocity and bidirectional partnership is included in grant agreements and memoranda of understanding.

With short funding cycles, and the typical insistence on “quick wins” and “low hanging fruits” from many funders, global health initiatives tend to be “surgical” as opposed to “organic” in their approach, resulting in superficial and short-lived initiatives that fail to sufficiently take the local context into account or have fundamental and sustained impact [3]. The danger of these “quick wins,” which come with their own agenda, accountability processes, and needs, is that they can shift local organisations’ processes away from their core goal.

Funders and donors need to be held accountable for building real, long-term, mutually beneficial, and reciprocal collaborations, with people on the ground in the driving seat, and a clearly defined shift in decision-making power on what is to be funded to local partners.

The persisting legacy of short-term funding is that it reproduces inequalities in local health systems in the form of vertical programming. The allure of the “surgical” is also there in how national governments in the global South shift in tandem to more tangible problems rather than those that require organic processes to tackle. It is seen in how the jobs, promotions, and fundability of academics in the global North (and increasingly in the global South) are based on tangible measures such as publications in high-impact journals and winning research grants, with much less (if any) focus on the ethics and (epistemic) justice implications of the work, the use of local knowledge, capacity building, or implementation.

Much of global health is conducted through universities and similar entities. It is a major problem that academics in the global North (and increasingly in the global South) are incentivised to focus primarily on personal development (e.g., tenure, awards, publications, and grants), often at the expense of real impact [48]. Universities and academic institutes involved in global health need to be held accountable for creating structures and processes that incentivise academics to be better allies (e.g., give credit to HIC and other privileged LMIC colleagues for their supportive, allyship work) and be responsive to decision-makers on the ground, engage with organic local processes and Indigenous knowledge, and engage with local partners as leaders of the process of knowledge production and use.

LMIC governments and institutions must invest more in their own healthcare delivery, research, and training, in order to reduce their dependence on HIC donors, universities, and philanthropies. Building quality research and teaching institutions in LMICs is critical, to reduce reliance on HICs and to improve the overall quality, depth, and relevance of scientific training and research [31].

### Phased self-decentralisation

We cannot reform global health without interrogating the very idea of global health itself, its underlying values, and even its vocabulary [1,2]. We need to understand the ways in which the colonial legacies deeply entrenched in national and global health systems impede the achievement of health equity.

The current global health landscape is heavily centralised and homogenous. Global health remains much too centred on individuals and agencies in HICs. Most “renowned” global health leaders are White, able-bodied men with a degree from an elite Western university, who lead organisations headquartered in the global North with ground operations governed from a distance [4]. A representative heterogeneous leadership and a decentralised mode of governance and operation are long overdue.

We would ideally hope that those at current global North centres of global health power will respond to our calls to decolonise global health by shifting power to people on the margins and the periphery [42].

But we cannot rely on this to happen by itself without a sustained push or demand. As Lioba Hirsch wrote, to be taken seriously, any commitment from global health institutions to undoing colonialism and fighting racism must be matched by demonstrated willingness “to give up some or all of their power” and “a radical redistribution of funding away from HICs, a loss of epistemic and political authority, and a limitation to [their] power to intervene in LMICs” [14].

It is never easy for HIC organisations or any other privileged group or individual to give up their power. We need commitments from global health organisations to which they can be held accountable. These organisations (e.g., universities and other academic institutes, philanthropic organisations, humanitarian organisations, and the academic publishing industry that publish in fields related to global health) need to recognise the consequences of being centralised and homogenous entities and take clear steps to become diverse and decentralised.

In particular, and as an example, universities and other institutes involved in global health research and training need to be held accountable for creating more opportunities for global health education and training which are designed, conducted, and imparted locally and are responsive to local contexts [49,50]. These institutes, especially schools of global public health, need to commit to being held accountable for perpetuating colonial and exploitative practices—e.g., in the form of Masters in Global Health programs which are so expensive that they are apparently not designed for people in LMICs or without privilege and for research training programmes that are designed primarily for students in the global North [51].

The global health classroom is now the world, and global health courses in HICs can use the virtual format to amplify voices from the Global South, Indigenous scholars, and BIPOC individuals with lived experience of oppression and resilience [52]. Remote teaching can be used to reach wider and diverse audiences, including groups that may not be enrolled in traditional degree programs [52].

Universities and academic institutes in the global North need to commit to decentralising their global health operations, by moving and spreading their current global North base to different locations across the global South, with ownership subsequently transferred too. They may fold their operations into global public health education and training institutes in the global South or expand their field-based faculty so that LMIC scientists can stay at home and work domestically—arrangement made much easier due to remote learning driven by COVID-19 [52]. Their operations in the global North may become minor or even cease to exist, thus helping to shift the centre of global health to the periphery. However, in doing so, they must avoid recreating themselves, but instead enable varying entities that speak to local circumstances in different parts of the world.

## Reimagining global health

The COVID-19 pandemic has been devastating for the entire world, with more than 125 million people affected and over 2.7 million dead as of March 2021. But the pandemic has also had a wider impact on all other areas of health, care, and global health. Years of progress in many areas of global health (e.g., immunisation, tuberculosis [TB], AIDS, and malaria) have been erased in a span of 1 year [53]. To make matters worse, the world is currently in the deepest global recession since the Second World War. As millions are pushed into poverty, health outcomes can only get worse. And, we still have the climate crisis looming in our immediate future.

As the current economic inequities get worse, and the privileged become more privileged, our collective ability to deal with these cumulative threats will be greatly diminished [54]. Indeed, the COVID-19 pandemic is a watershed moment in history. Are we going to continue the same path of widening inequities, where a small number of people own as much wealth as half the world’s population [55]?

Can global health be equitable when the world itself is not? The COVID-19 pandemic calls for a more equitable world and a new approach to global health research, education, and practice [54]. It calls for a decolonised and decentralised global health, one that moves beyond tokenistic box ticking about diversity and inclusion into developing new structures and processes that can address power asymmetries.

The decolonising global health discourse is generating a lot of interest, but we are just seeing the tip of the iceberg. Championed by predominantly young, BIPOC students, the discourse is challenging the status quo—and the skewed ways in which global health is being studied, taught, funded, researched, driven, designed, and implemented. We recognise that the accountability and reshaping of power dynamics are at the heart of all our proposals for change by ourselves and by organisations. These are not easy, even for people and organisations that are avowedly well intentioned and equity focused. But good intentions are not enough.

We are aware that the global health of our dreams and our wish list are unrecognisable from the global health of today. Much will have to change. But change is possible—if we are all willing to deepen our consciousness, listen deeply, listen differently, embrace global solidarity, and fight supremacy in all its forms. We are optimistic and hopeful that these dreams will become reality, since the COVID-19 crisis has made it imperative that humanity builds a more just and equitable world [54]. Fighting for such a world, during and after the pandemic, should be synonymous with global health practice.

**Disclaimers:** Kenneth Munge declares that the views expressed in this paper are entirely those of the author. They do not necessarily represent the views of the International Bank for Reconstruction and Development/World Bank and its affiliated organisations or those of the Executive Directors of the World Bank or the governments they represent.

## Abbreviations

COVID-19: Coronavirus Disease 2019
HIC: high-income country
LMIC: low- and middle-income country
