# Correction: Addressing power asymmetries in global health: Imperatives in the wake of the COVID-19 pandemic

**DOI:** 10.1371/journal.pmed.1003667

**Published:** 2021-06-07

**Authors:** The PLOS Medicine Staff

The byline for Cristian Montenegro is misspelled in the downloadable PDF for this article. The correct first name is Cristian. The publisher apologizes for the error.

## References

[1] Abimbola S, Asthana S, Montenegro C, Guinto RR, Jumbam DT, Louskieter L, et al. (2021) Addressing power asymmetries in global health: Imperatives in the wake of the COVID-19 pandemic. PLoS Med 18(4): e1003604. doi: 10.1371/journal.pmed.1003604 33886540 PMC8101997

